# Children’s and Parents’ Marburg Sugar Index (MSI) Values: Are They Comparable?

**DOI:** 10.3390/nu14081630

**Published:** 2022-04-14

**Authors:** Peter Schmidt, Andreas G. Schulte, Jutta Margraf-Stiksrud, Monika Heinzel-Gutenbrunner, Klaus Pieper

**Affiliations:** 1 Department of Special Care Dentistry, Witten/Herdecke University, Alfred-Herrhausen-Strasse 50, D-58448 Witten, Germany; andreas.schulte@uni-wh.de; 2 Department of Psychology, Philipps University of Marburg, Gutenbergstrasse 18, D-35032 Marburg, Germany; margrafs@staff.uni-marburg.de; 3 MH Statistik Beratung-Marburg, Bienenweg 8, D-35041 Marburg, Germany; monika.heinzel@mh-statistik.com; 4 Centre for Dentistry, Oral and Maxillofacial Medicine, Philipps University of Marburg, Georg-Voigt-Strasse 3, D-35033 Marburg, Germany; profpieper@t-online.de

**Keywords:** caries experience, living environment, children’s nutritional behaviour

## Abstract

Studies on children’s nutritional behaviour (CNB) rarely compare children’s answers regarding the frequency of their sugar intake with the respective statements of their parents. Therefore, data from a prevention study were used to analyse this aspect, as well as a potential correlation between Marburg Sugar Index (MSI) values and caries experience of children. The present study based its questionnaire data on CNB and caries data. Pairs of questionnaires filled in separately by children and parents of the participating families were dichotomised by either having completed the diet section entirely (Group A) or in part (Group B). The MSI scores were calculated separately for children and parents. The statistical confidence level was set at α = 0.05 (two-sided). Furthermore, the Pearson correlation coefficient was calculated and tested for “r = 0”. Additionally, a test for equality of the correlations was applied. The number of available questionnaire pairs was 429 in Group A and 400 in Group B. In both groups, significant correlations between children’s and parents’ MSI scores (A: r = 0.301, *p* < 0.001; B: r = 0.226, *p* < 0.001) were found. Using Spearman’s Rho, a significant correlation between MSI scores and children’s caries experiences was observed in Group A. MSI scores based on dietary questionnaires can be used to obtain consistent information on children’s CNB provided by the children themselves or their parents. This is true even when the MSI score has to be calculated on the basis of incomplete questionnaires. Questionnaire-based CNB information can improve the effectiveness of individual or group preventive measures supplemented by individually adapted nutritional counselling.

## 1. Introduction

Different authors have labelled caries as one of the most commonly occurring childhood diseases [1], if not even the most widely distributed chronic disease across the globe [2]. Moreover, the results of various studies indicate a linear correlation between the consumption of sugar and caries experience [3,4,5,6]. Nowadays, this correlation has become less apparent and is no longer deemed unconditionally true due to the effect of different caries-preventive confounders, such as fluoridation interventions or the use of fissure sealants in children and juveniles, which exert a moderating effect [7,8].

It is well-known that certain cariogenic oral bacteria, such as Streptococcus mutans, metabolise mono- and disaccharides. To be able to cause carious lesions, cariogenic microorganisms such as these must, however, have contact with tooth surfaces for a sufficient length of time [9,10]. The development of caries is thus promoted when orally ingested sugar interacts with bacterial plaque on tooth surfaces [11].

The large amounts of sugar consumed across the globe during the last several decades indicate that this threat to dental health is universal [6,12,13,14]. Moreover, sugar consumption also has a dire effect on health economics: In terms of economic costs in the year 2010, mono- and disaccharide-related dental diseases were associated with a global financial burden of 172 billion USD, the largest share of which (151 billion USD) was incurred in OECD countries [15]. Consequently, strategies designed to change dietary habits—in particular, a reduction of sugar intake—are gaining attention in the field of dental science as an approach to preventing caries, and, therefore, to improving oral health. There is, hence, a need for more clinical trials to assess the effectiveness of interventions based on psychological theory on reducing the dietary sugar intake in children, adolescents, and adults, as Al Rawahi pointed out [16].

To be able to precisely plan and execute appropriate individual or group preventions, it is, amongst other things, necessary to first collect reliable data on nutritional behaviours. In particular, in regard to children, the informative value of such surveys conducted to determine children’s nutritional behaviours (CNB) is, however, often limited. Chi et al. (2015), for example, reported that it is problematic to accurately capture the dietary habits of children and adolescents—in particular, in regard to their intake of sugar [17]. This problem, in part, stems from the fact that it is unclear whether children’s own responses to the CNB or those of their parents deliver more valid results.

Although various tools have been developed to survey children and adolescents in this context, the publications on the topic frequently fail to disclose whether the results were based on responses given by the children themselves or on responses given by their parents [18,19,20,21]. Moreover, to date, hardly any studies have focused on surveying both children and their parents to compare their responses in regard to CNB. Therefore, the present study set out to investigate to what extent children’s responses to a questionnaire surveying the frequency of their dietary sugar intake (the Marburg Sugar Index, MSI) correlated with their parents’ responses to the same questionnaire reporting about their children’s nutritional behaviour. An additional goal was to investigate whether correlations diverged for families from different social classes. The following hypotheses were tested:

1. Children’s responses in regard to CNB and those of their parents correlate.
2. The sugar indices are correlated with the caries experience.

In addition, due to the expectation that parents and children in families with a higher socioeconomic status (SES) talk about nutritional behaviour and oral hygiene more frequently than families with a lower socioeconomic status, the following question was also investigated: Are the correlations between responses given by parent–child pairs in regards to CNB higher in families with higher SES than in lower-class families?

## 2. Study Population and Methods

This study evaluated data previously collected within the scope of a caries prevention study carried out in the years 2010–2012 in primary schools in Northern Hesse, Germany [22]. The data collected from the questionnaire and the final examination in this previous study formed the dataset that was statistically evaluated in the present study (data on caries experience, SES, and CNB).

In the original study, 43 out of 53 primary schools in the North Hessian District “Waldeck-Frankenberg” (Germany) had participated in the project. Twenty schools were chosen by lot to be included in an intensified prevention program based on toothbrushing with elmex^®^ gelée (fluoride content 12,500 ppm, elmex research/Colgate-Palmolive Europe sàrl, Therwil, Switzerland) under the supervision of a “Tooth Brushing Fairy” (TBF) at intervals of three weeks during school sessions (test group).

The study was approved by the ethics committee of the Faculty of Medicine, University of Marburg, Germany (file number: study 146/10, chairman: Prof. G. Richter). In addition, informed written consent was obtained from the parents of participating children before the start of the study. Schoolchildren whose parents had not given written consent were excluded from the study. This was the case in less than 1% of the schoolchildren. Pupils with severe physical or learning disabilities were also excluded.

To determine the caries experience and caries increment, a basic examination was performed between September 2010 and May 2011 in 7- to 8-year-olds and a final examination in the same schoolchildren (9- to 10-year-olds) between August and November 2012 by using the International Caries Detection and Assessment System (ICDAS) [23]. The dental examinations were performed by using plane mirrors, blunt periodontal probes, compressed air for drying the teeth, and artificial light. No radiographs were taken. Instead, fibreoptic transillumination was used.

Apart from dietary habits as the most important independent variable of interest, other factors such as dental health knowledge, dental health behaviour, and attitudes toward prevention were examined psychometrically.

The respective surveys with standardised questionnaires were carried out among the then 8- to 11-year-olds and their parents shortly before the final dental examination. The parents filled out their questionnaires at home. The schoolchildren, on the other hand, completed their questionnaires in the classroom under the supervision of their class teacher, the dental examiner, and a dental assistant. This supervision ensured that the children did not influence each other while answering the questions and that they could ask the adults for advice, if they did not understand a question.

The new diet questionnaire had been developed to examine dietary habits, with the aim of obtaining information based on actual behaviours concerning eating habits relevant to oral health [8]. In designing the CNB questionnaire, the following principles were adhered to: Structured questions were formulated (“How often do you eat…”) that were related to specific everyday eating situations, especially between main meals, which proved to be relevant for higher caries risk. The food items consumed in these situations should contain foods harmless, as well as detrimental, to dental health (cariogenic).

The situations included

- Breakfast;
- Between-meal snacks;
- Watching television;
- Eating on the go.

The frequency of consumption of the listed food items was grouped into five options (never, seldom, occasionally, often, and always), which were given point values from 0 to 4. All checked foods and beverages that were tooth friendly were given a score of 0 points during processing of the data, regardless of how often they were reportedly consumed. The other foodstuffs were given the point scores listed above. For each situation, item scores were counted as the sum of the point values of the indicated foods. The number of food items varied between 7 and 11 in different situations. The questionnaire thus contained about 60 possible answers. The questionnaires were also used at earlier points in the period of a previous study and have, therefore, been described in depth in this context [24].

For further analyses, the item sum scores for all the situations were added to yield the so-called sugar index. This index serves as an indicator of the frequency of mono- and disaccharide intake, and a high value of the sugar index stands for unfavourable eating habits. The calculation of the “Marburg Sugar Index” (MSI) has also been described in earlier papers, where it was still called the “New Sugar Index” [8,22] The new diet questionnaire and a description for calculating the “Marburg Sugar Index” (MSI) can be found as online Supplementary. The link can be found behind “Supplementary Materials”.

In one of these papers, Winter et al. [22] also described the dichotomisation of the surveyed families into different SES subgroups. Three different indications for the parents were surveyed separately to calculate the SES and the respective characteristics of the variables coded in ascending order:

- Schooling: ranging from no diploma up to high school diploma enabling university attendance (1–5 points);
- Occupational training: ranging from no degree up to a university degree (1–4 points);
- Occupational status: ranging from unemployed up to academic/self-employed (1–6 points).

The SES data were obtained separately for father and mother and combined into a total score. Within a couple, the higher professional position was evaluated. If there was only one parent in the family, or if the information of a partner was missing, the existing information was used, whereby the determined value was doubled. The range of the SES scores was 6–30. Thus, it was possible to dichotomise the parents into two different subgroups according to their SES:

- Low class (Low SES): 6–18 points;
- Middle and upper class (Higher SES): 19–30 points.

Further detailed information on the study area and study population can be found in previously published papers [8,22].

### Statistical Analysis

Statistical analysis for the present study was performed using IBM^®^ SPSS Version 24 software (IBM, Armonk, NY, USA). The confidence level for the significance tests with two-sided testing was α = 0.05. The Pearson correlation coefficient r was calculated and tested for “r = 0” as a measure of the linear relationship between the MSI score determined on the basis of the children’s or parents’ data. Furthermore, a test for equality of the correlation was performed, where N was the testing variable for the difference between two correlations. The association between the sugar indices and the caries experiences was evaluated by Spearman’s Rho.

## 3. Results

In total, 1089 fourth graders had filled out children’s questionnaires, while 829 filled-out parent questionnaires, which had been taken home, were returned. During the evaluation, it turned out that some of the participants in the survey did not respond to all of the food items. For this reason, two groups were formed for the statistical analysis:

- Group A comprised matched parent–child pairs who had responded to all of the food items in the diet section of the questionnaire.
- Group B comprised matched parent–child pairs who did not respond to all of the food items in the diet section of the questionnaire.

**Figure 1:**
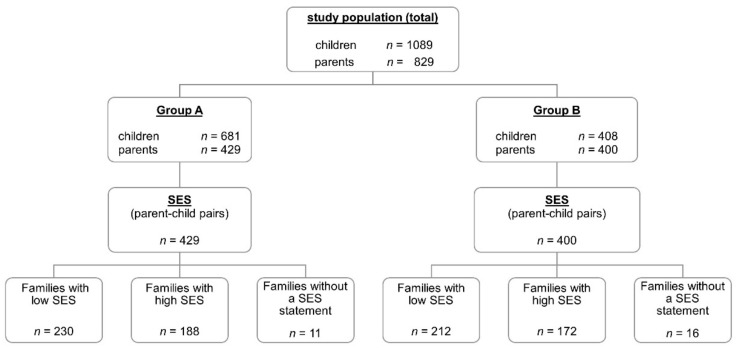
Overview of the study population (children and parents) and the distribution among the different subgroups (Group A—questionnaires with a fully completed diet section; Group B—questionnaires with incompletely completed diet sections; SES—socioeconomic status).

Details on the studied collective and the distribution thereof in the various subgroups are presented in Figure 1 and Table 1.

Data from Group A, in which 681 children’s questionnaires and 429 parents’ questionnaires were available, were used to test our hypotheses. In this group, the questionnaires for 429 parents and their children could be matched to yield a total of 429 parent–child pairs for comparison. Based exclusively on the responses of the 429 schoolchildren belonging to these parent–child pairs, a mean MSI score of 48.6 was calculated for their nutritional behaviours (Table 2). A mean MSI score of 49.8 was calculated based exclusively on the responses from the 429 parental questionnaires. The distributions in each of these two subgroups did not deviate significantly from the normal distribution. The MSI scores for the two subgroups were, however, significantly correlated (r = 0.301/*p* < 0.001). Further statistical values for Group A are given in Table 2.

To gain a more complete picture, the results for Group B, in which the questionnaires were not wholly completed, were similarly evaluated. In this group, 408 children’s questionnaires and 400 parents’ questionnaires were available. Additionally, in this evaluation, only questionnaires that were filled out by both parent and child were considered (i.e., *n* = 400 parent–child pairs).

Table 3 shows the frequencies for the individual items omitted in the children’s and in the parents’ diet questionnaires. As can be seen, in 85% of the cases, the participants omitted no more than two items. There was thus still sufficient data to also estimate the correlation between the parent–child responses in Group B. The correlation between the MSI scores determined from the children’s questionnaires and those determined from the parents’ questionnaires was also found to be significant in this group. The correlation coefficient for this group was r = 0.226 (*p* < 0.001). The test for equality of the correlation showed no statistically significant difference between the two correlations (N = 1.156, *p* = 0.124).

### 3.1. Correlation between the Children’s Caries Experience and the Sugar Indices of the Parent–Child Pairs

**Figure 2:**
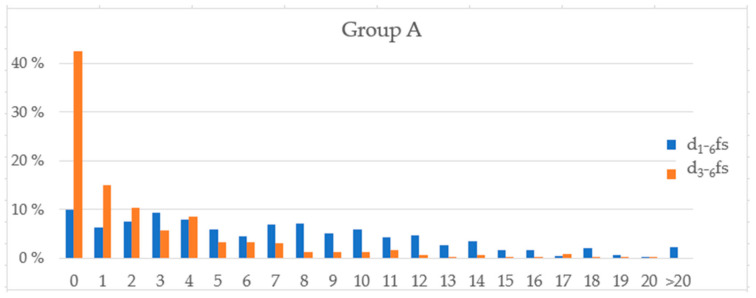
Distribution of the dfs scores (d_1–6_fs and d_3–6_fs) for schoolchildren from Group A.

Figure 2 shows the distribution of the dfs scores for schoolchildren from Group A.

Table 4 shows the correlation between the sugar scores determined from the children’s responses and the caries scores d_1_–_6_fs and d_3_–_6_fs for Group A, as well as for those determined from the parents’ responses. Three of the correlations were statistically significant.

### 3.2. SES and Correlations between the MSI Values for Parent–Child Pairs

#### 3.2.1. Group A

**Figure 3:**
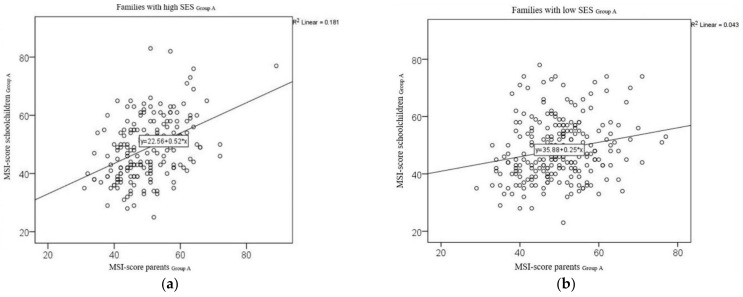
Correlation of the MSI scores for all parent–child pairs with different SES from Group A: (**a**) left—Group A, families with high SES; (**b**) right—Group A, families with low SES.

Four hundred and eighteen (418) parent–child pairs with fully answered questionnaires could be dichotomised into groups with higher and lower socioeconomic statuses. Of the 418 questionnaire pairs, 230 questionnaire pairs (53.6%) were from families belonging to a lower SES group (low SES), and 188 (43.8%) of the questionnaire pairs were from families belonging to a higher SES group (high SES). In 11 cases, parents did not disclose which social class the family belonged to (Figure 1 and Table 5). The correlation coefficient for the MSI values for the parent–child pairs from lower SES families was r = 0.206 (*p* = 0.002) and r = 0.425 (*p* < 0.001) for those from higher SES families. The test for equality of the correlation showed a statistically significant difference between the two correlations (N *=* 2.471, *p* = 0.013). Figure 3 illustrates the correlations between the MSI scores for the parent–child pairs in Group A for both status classes in scatterplots.

#### 3.2.2. Group B

The correlation coefficient for the MSI values for the parent–child pairs in Group B was r = 0.212 (*p* = 0.002) for families with low SES and r = 0.225 (*p* = 0.003) for families with high SES. The test for equality of the correlation showed no statistically significant difference between the two correlations (N *=* 0.132, *p* = 0.895).

Depending on how fully the questionnaires were completed, the correlation between the MSI scores for the children and those of the parents was, thus, more marked for families with high SES than for families with low SES (Figure 3 and Table 5). This was true for Group A but not for Group B.

## 4. Discussion

In an earlier study, Pieper et al. already gained important insights in regards to the use of their newly developed nutrition questionnaire [8]. The researchers reported that the questionnaire, which was used as a data collection tool, “(a) gave a good depiction of eating habits relevant to dental health, as the correlation with caries experience showed; (b) showed a good acceptance for children and (c) that a content validity can be claimed”. The results of the current study, which were based on data acquired in the context of Winters et al.’s study [22], have yielded even further insights. In light of the fact that the correlation between the children’s answers and those of their parents in respect to CNB was found to be significant, Hypothesis 1 of our study can thus be accepted. Similarly, our results also corroborate Hypothesis 2.

The finding that both the children and their parents provided correlating information in respect to CNB is important for several reasons. On the one hand, this shows that parents do seem to have a good idea of their children’s CNB, even if the correlation between their responses and those of their children was not strong enough to suggest that one of these responders might be replaced by the other. On the other hand, this confirms that predictions about caries experience and caries increment are possible both on the basis of CNB data given by the children, as well as on the basis of that given by their parents.

Moreover, the results show that the correlation between MSI values in parent–child pairs was similar, regardless of whether the calculations of the sugar indices were based on completely or on incompletely answered questionnaires. This finding suggests that the level of sugar consumption can be confidently estimated even if a few items in the questionnaire are omitted.

Furthermore, as the evaluation of the results shows, the MSI scores in regard to CNB correlated more strongly within parent–child pairs in families with high SES than in families with low SES. Our second hypothesis, thus, is also confirmed.

To date, the published literature has consisted mainly of studies based on CNB questionnaire surveys that were conducted exclusively on groups of children, adolescents, or adults [18,19,20,21,25]. However, the validity of surveys conducted exclusively on children is a controversial topic. In this respect, the ages of the surveyed children must be considered a decisive factor.

Since children’s cognitive ability to self-report on the foods they eat only begins to rapidly increase from the ages of 7 to 8 years onwards, parents have been found to be the more reliable reporters of their child’s food intake in children younger than this [26]. From the ages of 10 to 12 years onward, children are then generally able to reliably report their own food intake; however, it should be borne in mind that, in adolescents, self-reports in this respect can be subject to distortion, due not only to less interest in this age group in participating in surveys of this kind, but also to the influence of peer groups or even social desirability [26].

Although the correlation between the MSI scores from the children’s questionnaires and the parents’ questionnaires was found to be only moderate in our study, it was, nonetheless, still significant. In Group A, the mean values for these scores were close together (Table 1). For some of the parent–child pairs in this group, the responses given by the children and their parents diverged considerably on an individual basis. This finding can also be seen in the corresponding scatterplots: Some of the MSI values calculated on the basis of the children’s questionnaires were almost twice as high as the MSI scores calculated on the basis of their parents’ questionnaires (Figure 3).

Determination of the MSI score is important in terms of being able to offer and implement individual measures in dental practices. Since 1993, statutory health insurances in Germany, in which around 90% of the population are enrolled, have included various individual prophylactic dental measures in the scope of their services [27,28]. In 2019, the scope of this service was further expanded to also include dental screening programs for young children [29]. In Germany, every child aged between 6 months and 17 years is thus entitled to a range of preventive services that also encompass dietary oral health counselling by a dentist [30]. Guidance for dentists on how best to assess the dietary habits of their patients has, however, been lacking so far.

Our questionnaire is fundamentally a suitable tool for this purpose, as it allows the recording of dietary intake in everyday situations, and the responses to the included questions can serve as a basis from which to determine the counselling needs of patients in regards to their current nutritional risks [8]. Pieper et al. (2019) previously identified various aspects that could help dentists use both the questionnaire and the sugar index calculated on the basis of the responses sensibly within their daily clinical routine. The authors of that study offered the following suggestion: “The patients could be advised to reduce their sugar index to a value below the median, if they want to make a greater contribution to prevention by reducing their sugar intake” [8].

As the results of the present study show, the responses of the 8- to 11-year-olds who participated in the survey were nearly as reliable as those given by their parents. This insight is corroborated by the fact that no significant differences were found between the correlations (Table 4). Livingston and Robson also emphasised in their publication that parents are usually well able to report on their children’s CNB at home [26].

As Pieper et al.’s (2019) previously published article on MSI reported, parents considerably influence their children’s CNB [8]. In that study, a significant correlation between caries experience and sugar consumption was found for the deciduous teeth of fourth graders. This observation led the authors to conclude that caries experience in deciduous teeth was largely related to the dietary habits of families.

As can be seen in Figure 2, the deviation in the MSI scores between parents and children in the parent–child pairs from lower social classes was greater than that for the parent–child pairs from higher social classes. This finding may be related to differences in dietary behaviours in different social settings. Further independent variables that may act as confounding factors on the correlation between sugar consumption and caries experience have been described elsewhere [31,32,33,34,35,36,37,38]. Socioeconomic status (SES) is one of the named factors [22,24,35].

Winter et al. (2018) thus reported that the caries experiences of children from families with low SES are greater than those of children from families with high SES [22]. They, however, also reported that the early implementation of intensive prevention programs can help level out this initial disadvantage [22]. It is on the basis of these findings that Winter et al. deduced that group prophylaxis could prove to be a pertinent measure, especially in socially problematic settings (deprived areas and suburbs), as the prerequisite for individual prophylaxis in children is that they are taken to a dental practice or dental clinic. As various other authors have also pointed out, visits not only to dentists but, also, to paediatricians may pose barriers to the acceptance of preventive measures in families with low SES [31,39]. A study conducted by Hong et al. (2018) illustrated that children with a low social status not only consume more sugar than children with a high social status, they also visit the dentist less frequently [31].

Children from families in which both parents hold out-of-home jobs frequently attend full-time schools or day-care centres or are looked after by other persons, such as grandparents or childminders. Our current study did not include a survey of persons from these groups, but it might prove interesting to also survey persons from the children’s wider circle of care providers in future studies on CNB.

## 5. Conclusions

The dietary questionnaires used in this study are a tool to gather data on CNB. Since the reliability of the responses children give to the items on the questionnaire depends on the child’s age, it is advisable to also include parents or other caregivers from the child’s social circle in the survey, especially for young children. The results of the present study corroborate that the used questionnaire is suitable for surveys in various groups (i.e., children and their parents) and delivers interpretable results. Both the responses given by the children and by their parents proved to be a suitable means to portray relevant dietary behaviours that predict oral health. The questionnaire and the MSI score determined from the responses can thus be used as a tool in everyday practice by dental professionals, such as regular dentists, dental hygienists, or dentists working for the public health services. This gain in information on CNB can help increase the effectiveness of individual or group preventive measures and can help the dentist custom tailor dietary advice to the patient’s individual needs.

## Figures and Tables

**Table 1:** Demographic description of schoolchildren from the parent–child pairs of Group A and Group B (gender and age distributions).

	Gender		Age Distribution				Total
	Boys	Girls	8 yrs	9 yrs	10 yrs	11 yrs	
**Group A**							
*n*	216	213	28	307	91	3	429
	50.3%	49.7%	6.5%	71.6%	21.2%	0.7%	100%
**Group B**							
*n*	187	213	26	281	85	8	400
	46.8%	53.2%	6.6%	70.3%	21.3%	2.0%	100%

**Table 2:** Mean values of the MSI scores of the children and parents from Group A.

	Children	Parents
*n*	429	429
MSI score (mean)	48.6	49.8
MSI score (min)	23	29
Median	48	49
MSI score (max)	83.0	89.0
±SD	10.4	8.6

The maximum possible value of the MSI is 125.

**Table 3:** Frequencies of missing data for children and parents (Group B) in the diet section of the questionnaire (number of items was 22).

Number of Omitted Items	Children (%)	Parents (%)
0–1	70.0	80.8
2	13.5	7.5
3	5.5	2.3
4–5	5.3	5.0
6 and more	5.7	4.5

**Table 4:** Correlations of the MSI scores calculated from the parents and children’s responses with the outcome variables d_1–6_fs and d_3–6_fs from Group A.

	d_1_–_6_fs		d_3_–_6_fs	
	MSI Score (Children)	MSI Score (Parents)	MSI Score (Children)	MSI Score (Parents)
*n*	429	429	429	429
Spearman’s Rho	0.107	0.111	0.053	0.111
*p*-value	0.026 *	0.021 *	0.275	0.022 *

* Statistically significant.

**Table 5:** Correlation of the MSI scores for all parent–child pairs with different SES from Group A and Group B.

	Total	Low SES	High SES
**Group A**			
*n*	429	230	188
r-value	0.301	0.206	0.425
*p*-value	<0.001 *	0.002 *	<0.001 *
**Group B**			
*n*	400	212	172
r-value	0.226	0.212	0.225
*p*-value	<0.001 *	0.002 *	0.003 *

* Statistically significant.

## Data Availability

Not applicable.

## Supplementary Materials

The new diet questionnaire and a description for calculating the “Marburg Sugar Index” (MSI) can be found as online Supplementary Files S1 and S2 (see www.karger.com/doi/10.1159/000486102 for all online Supplementary Materials).
